# Inhibitory Growth of Oral Squamous Cell Carcinoma Cancer via Bacterial Prodigiosin

**DOI:** 10.3390/md15070224

**Published:** 2017-07-15

**Authors:** Ming-Fang Cheng, Chun-Shu Lin, Yu-Hsin Chen, Ping-Jyun Sung, Shian-Ren Lin, Yi-Wen Tong, Ching-Feng Weng

**Affiliations:** 1 Department of Pathology, Tri-Service General Hospital, National Defense Medical Center, Taipei 10086, Taiwan; drminfung@gmail.com; 2 Division of Histology and Clinical Pathology, Hualian Army Forces General Hospital, Hualien 97144, Taiwan; 3 Department of Radiation Oncology, Tri-Service General Hospital, National Defense Medical Center, Taipei 10086, Taiwan; Chunshulin@gmail.com; 4 Department of Life Science and Institute of Biotechnology, National Dong Hwa University, Hualien 97401, Taiwan; 810013103@gms.ndhu.edu.tw (Y.-H.C.); pjsung@nmmba.gov.tw (P.-J.S.); d9813003@gms.ndhu.edu.tw (S.-R.L.); mecurry@gmail.com (Y.-W.T.); 5 Graduate Institute of Marine Biotechnology, National Dong Hwa University, Pingtung 94450, Taiwan; 6 National Museum of Marine Biology and Aquarium, Pingtung 94450, Taiwan

**Keywords:** prodigiosin, marine viva, autophage, oral squamous cell carcinoma

## Abstract

Chemotherapy drugs for oral cancers always cause side effects and adverse effects. Currently natural sources and herbs are being searched for treated human oral squamous carcinoma cells (OSCC) in an effort to alleviate the causations of agents in oral cancers chemotherapy. This study investigates the effect of prodigiosin (PG), an alkaloid and natural red pigment as a secondary metabolite of *Serratia marcescens*, to inhibit human oral squamous carcinoma cell growth; thereby, developing a new drug for the treatment of oral cancer. In vitro cultured human OSCC models (OECM1 and SAS cell lines) were used to test the inhibitory growth of PG via cell cytotoxic effects (MTT assay), cell cycle analysis, and Western blotting. PG under various concentrations and time courses were shown to effectively cause cell death and cell-cycle arrest in OECM1 and SAS cells. Additionally, PG induced autophagic cell death in OECM1 and SAS cells by LC3-mediated P62/LC3-I/LC3-II pathway at the in vitro level. These findings elucidate the role of PG, which may target the autophagic cell death pathways as a potential agent in cancer therapeutics.

## 1. Introduction

Oral cancer is the main cause of cancer death in males in Taiwan and is ranked the fourth leading cause of death overall. Oral cancer mortality and incidence data show that men with oral cancer increases annually. This trend derives from men smoking cigarettes, drinking alcohol, and chewing betel nut. Surprisingly, when a human subject has all three of these habits, the relative risk of oral cancer increases by 122.8 times [1]. Oral squamous cell carcinoma (OSCC) is common in both genders, followed by verrucous carcinoma, undifferentiated carcinoma, and small salivary adenocarcinoma. OSCC is a common type of head and neck cancer [2], excluding oropharynx and hypopharynx. OSCC is locally destructive, may invade the soft tissue and bone, and can be extended to nerves, lymphatic system, and blood vessels. Through these mechanisms, it can spread throughout the body and cause cervical lymph nodes metastasis and distant metastasis [3]. To avoid burdens of the chemotherapy agent in cancer patients, the inductions of cell apoptosis and autophagy are taken firstly into consideration during therapy regimen. This theme is becoming the critical standard for anti-cancer drug discovery.

Autophagy has been shown to be an intracellular degradation process in eukaryotic cells in response to stress, including starvation, clearing damaged proteins and organelles, and promoting cell survival [4,5]. Furthermore, autophagy occurs in multiple processes including nucleation, expansion, and maturation/retrieval to exert the effects of either autophagic cell death or cytoprotection [6]. Currently, two pathways have been investigated to associate with the regulation of autophagy in mammalian cells including the PI3K Class III/Akt/mTOR/p70S6K signaling pathway and the Ras/Raf/MEK/ERK1/2 pathway [7,8]. The ERK1/2 pathway could positively activate autophagy, whereas the Akt/mTOR pathway might suppress autophagy. These signaling pathways could be activated in numerous tumors and are thought to trigger the autophagy and oncogenesis [9]. The stimulation of mTOR protein level is a central regulator of autophagy [10]. Moreover, Akt was associated with cell survival and its expression could down-regulate the LC3-II expression; thereby, suppressing autophagy [11,12,13]. The amount of LC3-II is demonstrated to correlate well with the amount of autophagosomes [1,14].

Prodigiosin (PG, PubChem CID: 5377753), an alkaloid and natural red pigment, that is a secondary metabolite of *Serratia marcescens*. It is characterized by a common pyrrolyl pyrromethene skeleton [15,16]. Bacterial PGs and their synthetic derivatives have antimicrobial (bactericidal and bacteriostatic) [17,18,19,20], antimalarial [17,18,21], and antitumor [17,18,22,23,24] properties. Additionally, they have been shown to be effective apoptotic agents against various cancer cell lines [25], with multiple cellular targets, including multi-drug resistant cells with little or no toxicity towards normal cell lines, and induce apoptosis in T and B lymphocytes [26,27]. Moreover, PG and its structural analogue (compound R) have induced the expression of p53 target genes accompanied by cell-cycle arrest and apoptosis in p53-deficient cancer cells [28]. PG could be effective as a potential inhibitor compound against COX-2 protein, and can be applied as an anti-inflammatory drug [29]. In melanoma cells, PG activates the mitochondrial apoptotic pathway by disrupting an anti-apoptotic member of the BCL-2 family-MCL-1/BAK complexes by binding to the BH3 domain [30]. Additionally, PG exerts nearly identical cytotoxic effects on the resistant cells in comparison to their parental lines, revealing that this pro-apoptotic agent acts independently on the overexpression of multi-drug resistance transporters-MDR1, BCRP, or MRP [31]. Mechanistically, PG engages the IRE1-JNK and PERK-eIF2α branches of the UPR signaling to up-regulate CHOP, which in turn mediates BCL-2 suppression to induce cell death in multiple human breast carcinoma cell lines [32].

Anti-cancer chemical drugs for oral cancer include 5-FU, cisplatin, paclitaxel, and Ufur. Nonetheless, these chemotherapy drugs always induce side effects, such as nausea, vomiting, loss of appetite, decreased immunity, and adverse effects of oral ulcers. Recently, many natural sources [33,34,35,36,37] and traditional medicinal herbs [38,39,40] have been studied on OSCC in an effort to mitigate the abovementioned problems, however the solicitation remains incompletely explored. The present study investigated whether PG could have benefits to cause the inhibiting growth of human gingival squamous carcinoma cells (OECM-1) and human tongue cancer (SAS) cell lines by 3-(4,5-Dimethylthiazol-2-yl)-2,5-diphenyltetrazolium bromide (MTT) assay, flow cytometry assay, Western blotting and autophagosome formation assay.

## 2. Results and Discussion

### 2.1. Effect of Prodigiosin on the Cell Viability of Oral Cancer Cells

**Figure 1:**
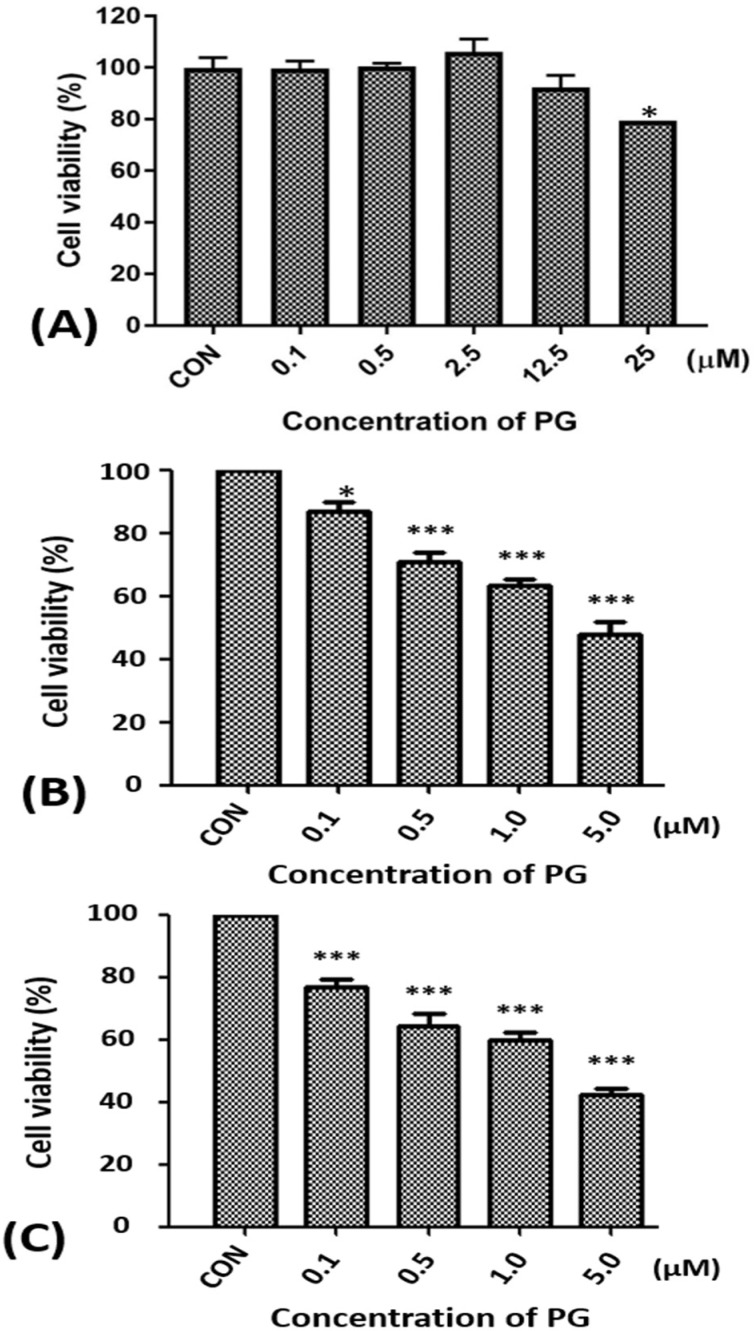
Alterations of the cell viability of FL83B, SAS, and OECM1 cells after prodigiosin treatment. (**A**) FL83B cells were incubated with 0.1 to 25 μM of prodigiosin (PG) for 24 h, (**B**) SAS and (**C**) OECM1 cells were incubated with 0.1, 0.5, 1.0, and 5.0 μM of PG for 24 h, and cell viability was determined by 3-(4,5-Dimethylthiazol-2-yl)-2,5-diphenyltetrazolium bromide (MTT) assay. The data are shown as the mean ± SEM of three independent experiments. * *P* < 0.05; *** *P* < 0.001 when compared with the untreated controls (0 μM).

To determine the cytotoxicity of PG in normal cell, normal mouse hepatocyte FL83B cell line was examined. The cell viability of FL83B with various concentration of PG treatment was found no significant cytotoxicity of PG treatment except less cytotoxicity at 25 μM (Figure 1A). In addition, SAS and OECM1 cells were initially treated with 0.1, 0.5, 1.0, and 5.0 μM of PG for 24 h to measure cell viability for the cytotoxic effect of PG. The viable SAS and OECM1 cells were significantly decreased after PG treatment in a dose-dependent manner (Figure 1B,C). The values of IC_50_ on OECM1 and SAS cells were also determined at the concentrations of 1.59 ± 0.77 and 3.25 ± 0.49 μM of PG, respectively. This result indicated that the low concentration of PG elicits the cytotoxicity of OSCC as compared to the untreated cells. Previous reports have demonstrated that PG could cause cell death via apoptotic cell death in several tumors, including human leukemia cells, melanoma, neuroblastoma, colorectal cancer, and breast cancer [32,41,42]. PG and its structural analogue (compound R) are also proven to induce cell-cycle arrest by the expression of p53 target genes in p53-deficient cancer cells [28]. Additionally, as p53 tumor suppressor integrated multiple stress signals, serial anti-proliferative responses would occur to induce apoptosis [43]. In this study, firstly, we presented the inhibitory growth role of PG in human oral squamous cell carcinoma cells in vitro and loss of cell viability was initially found in OECM1 and SAS cells with a dose-dependent fashion after 24 h incubation of PG (Figure 1). Moreover, the IC_50_ of PG on OECM1 and SAS cells were in the low and potential extent.

### 2.2. Effect of Prodigiosin on the Cell Cycle of Oral Cancer Cells

Obviously, cell cycle of SAS cells after 12 and 24 h of PG treatments showed different pattern. In 12 h treatments of PG, sub-G_1_ and S phase of SAS cells were not significantly different and G_0_/G_1_ phase of SAS cells raised from 40.3 ± 3.3% to 51.4 ± 1.2% (*P* < 0.05). G_2_/M phase of SAS cells was decreased from 32.4 ± 2.9% to 27.2 ± 0.7% (*P* < 0.05). In 24 h treatments of PG, S phase of SAS cells was still not significantly different but sub-G_1_ and G_0_/G_1_ phase of SAS cells were elevated from 0.9 ± 0.3% to 2.5 ± 0.7% and 42.1 ± 2.7% to 54.0 ± 3.7%, respectively (*P* < 0.05). G_2_/M phase of SAS cells was also decreased from 36.6 ± 2.1% to 26.3 ± 3.2% (*P* < 0.05; Table 1).

**Figure 2:**
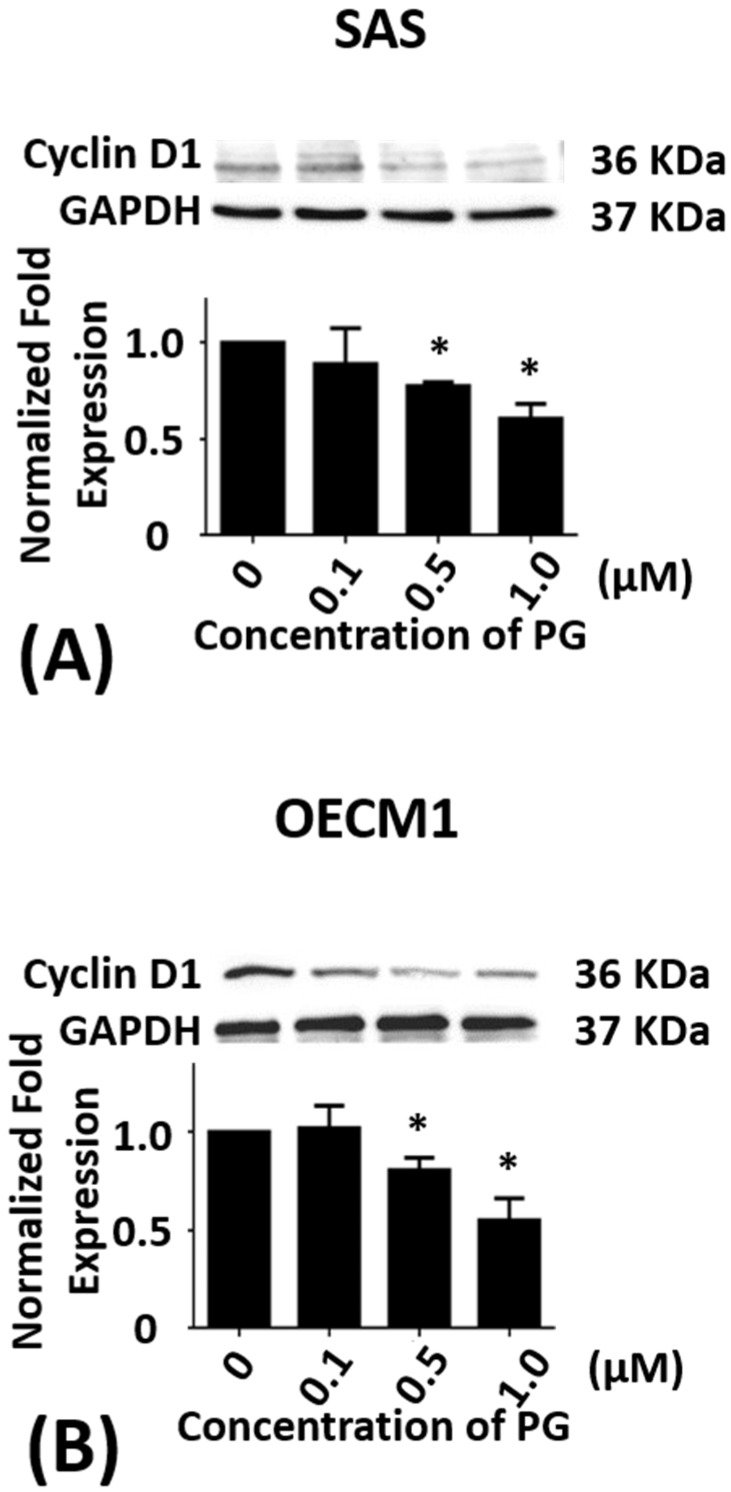
Altered protein levels of cyclin D1 of SAS and OECM1 cells treated with prodigiosin. SAS and OECM1 cells were treated with 0.1, 0.5, and 1.0 μM of prodigiosin (PG) for 24 h and lysed in RIPA buffer for Western blotting. Protein level of cyclin D1 in SAS (**A**) and OECM1 (**B**) cells were shown as the mean ± SEM of three independent experiments. Protein levels were represented as ratio of band intensity to untreated control, which were normalized via internal control GAPDH. * *P* < 0.05 when compared with the untreated control (0 μM).

As SAS cells, sub-G_1_ phase of OECM1 cells in 12 h treatments of PG were not significantly different but G_0_/G_1_ phase of OECM1 cells was significantly increased from 50.9 ± 1.7% to 63.3 ± 0.4% (*P* < 0.05). S and G_2_/M phase of OECM1 cells were decreased from 16.6 ± 1.0% to 10.5 ± 0.2% and 32.1 ± 0.4% to 25.7 ± 0.8%, respectively (*P* < 0.05). In 24 h treatments of PG, sub-G_1_ phase of OECM1 cells was not significantly different but G_2_/M phase of OECM1 cells was decreased from 36.9 ± 3.1% to 18.7 ± 3.3%, respectively (*P* < 0.05). G_0_/G_1_ and S phase of OECM1 cells were increased from 47.9 ± 2.3% to 61.8 ± 0.4% and 14.0 ± 1.6% to 18.4 ± 2.6%, respectively (*P* < 0.05; Table 2). The above results indicated that PG might inhibit cell growth via arresting cell cycle in G_0_/G_1_ phase. The protein level of cyclin D1 was analyzed to ensure the hypothesis of cell cycle arrest. Cyclin D1 in two cell lines was significantly decreased after 0.5 and 1.0 μM of PG treatments, which was consistent with the result of cell cycle analysis (*P* < 0.05; Figure 2A,B). These findings indicated that PG could induce cell cycle arrest and delay cell cycle progression, which attributed to inhibitory growth effects of PG in oral cancer cells. In addition, the cell cycle distribution after PG stimulation was observed to arrest in G_0_/G_1_ phase of SAS cells with various concentrations of PG treatment for 12 h, and in G_0_/G_1_ phase of OECM1 cells with various concentrations of PG treatment for 12 and 24 h. The findings demonstrated that PG could induce type II program (autophagy) cell death in these cancer cells in a time- and dose-dependent manner. Moreover, there was no significant change of sub-G_1_ level in OECM1 and SAS cells after 24 h treatment of PG. We also discovered GFP-LC3 puncta formation in PG-treated OECM1 and SAS cells, which indicated an increase of autophagosome formation in two oral cancer cells (data not shown).

### 2.3. Effects of Prodigiosin on AMPKα, PI3K Class III and Akt Protein Levels in Oral Cancer Cells

**Figure 3:**
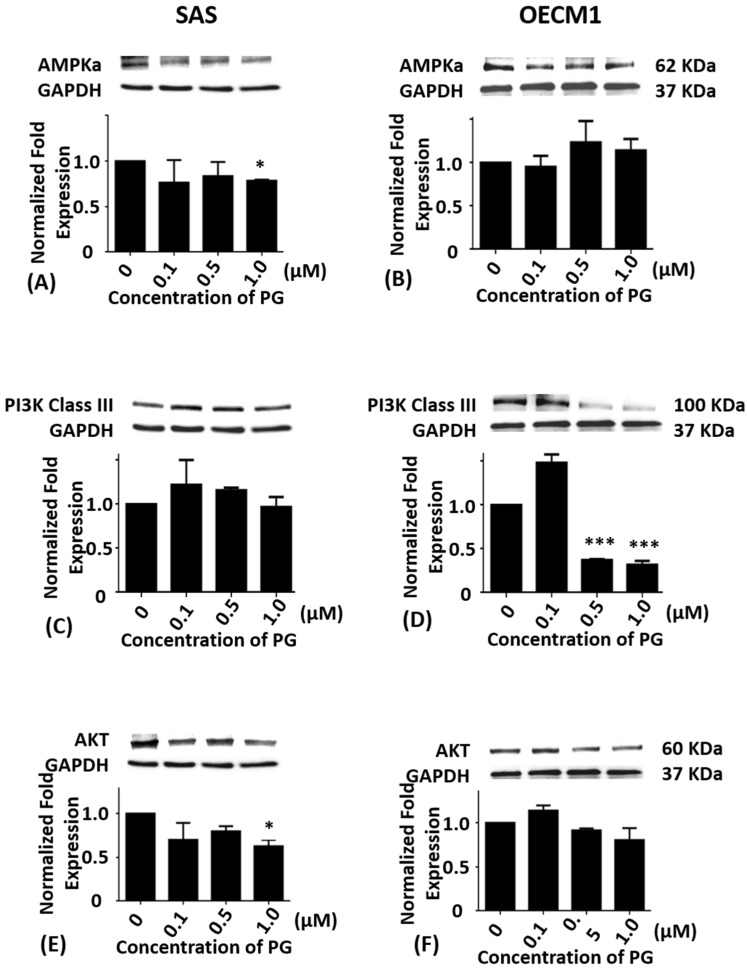
Altered protein levels of AMPKα, PI3K class III, and Akt of SAS and OECM1 cells treated with prodigiosin. SAS and OECM1 cells were treated with 0.1, 0.5, and 1.0 μM of prodigiosin (PG) for 24 h and lysed in RIPA buffer for Western blotting. Protein levels of AMPKα (**A**,**B**), PI3K class III (**C**,**D**), and Akt (**E**,**F**) were shown as the mean ± SEM of three independent experiments. Protein levels were represented as ratio of band intensity to untreated control which were normalized via internal control GAPDH. * *P* < 0.05 and *** *P* < 0.001 when compared with the untreated control (0 μM).

Cumulative studies have shown that autophagy is mediated by numerous signaling pathway including PI3K/Akt/mTOR [7,8], AMPK/mTOR/Ulk1 [44,45], and Beclin-1 [46]. To evaluate whether PG-induced cell death was related to autophagy, the autophagy-related protein levels of AMPKα, PI3K Class III, Akt, mTOR, Beclin-1, P62, LC3-I, and LC3-II in SAS and OECM1 cells were determined by Western blotting analysis. Compared with the untreated controls, the protein levels of AMPKα in SAS cells exhibited significant differences at 1.0 μM of PG treatment (*P* < 0.05; Figure 3A) while the protein levels of AMPKα in OECM1 cells showed no significant differences in various concentrations of PG treatment (Figure 3B).

When compared with the untreated control, the protein levels of PI3K class III in SAS cells showed no significance (Figure 3C). While the protein levels of PI3K class III in OECM1 cells were markedly down-regulated in 0.5 and 1.0 μM of PG treatments (*P* < 0.05; Figure 3D). The protein level of Akt in SAS and OECM1 cells were evaluated with 0.1, 0.5, and 1.0 μM of PG for 24 h treatments, the results revealed no significant alteration when compared with the untreated controls except significant decrease in 1.0 μM of PG in SAS cells (Figure 3E,F).

### 2.4. Effects of Prodigiosin on mTOR and Beclin-1 Protein Levels in Oral Cancer Cells

**Figure 4:**
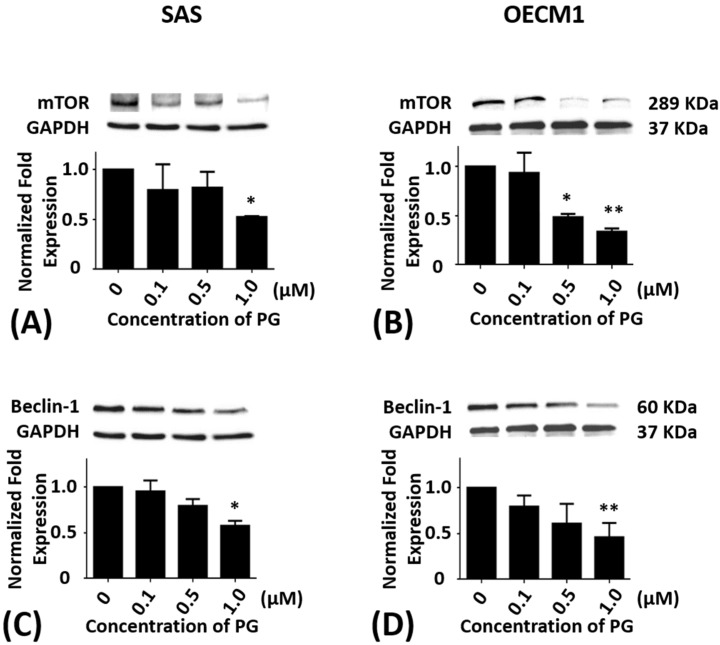
Altered protein levels of mTOR and Beclin-1 of SAS and OECM1 cells treated with prodigiosin. SAS and OECM1 cells were treated with 0.1, 0.5, and 1.0 μM of prodigiosin (PG) for 24 h and lysed in RIPA buffer for Western blotting. Protein levels of mTOR (**A**,**B**) and Beclin-1 (**C**,**D**) were shown as the mean ± SEM of three independent experiments. Protein levels were represented as ratio of band intensity to untreated control which were normalized via internal control GAPDH. * *P* < 0.05 and ** *P* < 0.01 when compared with the untreated controls (0 μM).

SAS and OECM1 cells were incubated with 0.1, 0.5, and 1.0 μM of PG treatment for 24 h. As compared with the untreated controls, the protein levels of mTOR in SAS cells were significantly decreased in 1.0 μM of PG treatment (*P* < 0.05; Figure 4A). The protein levels of mTOR in OECM1 cells were also reduced in 0.5 and 1.0 μM of PG treatments (*P* < 0.05; Figure 4B). After treatment with 0.1, 0.5, and 1.0 μM of PG for 24 h, the protein levels of Beclin-1 were significantly decreased in 1.0 μM of PG treatment in SAS and OECM1 cells when compared with the untreated controls (*P* < 0.05; Figure 4C,D).

### 2.5. Effects of Prodigiosin on P62, LC3-I and LC3-II Protein Levels in Oral Cancer Cells

**Figure 5:**
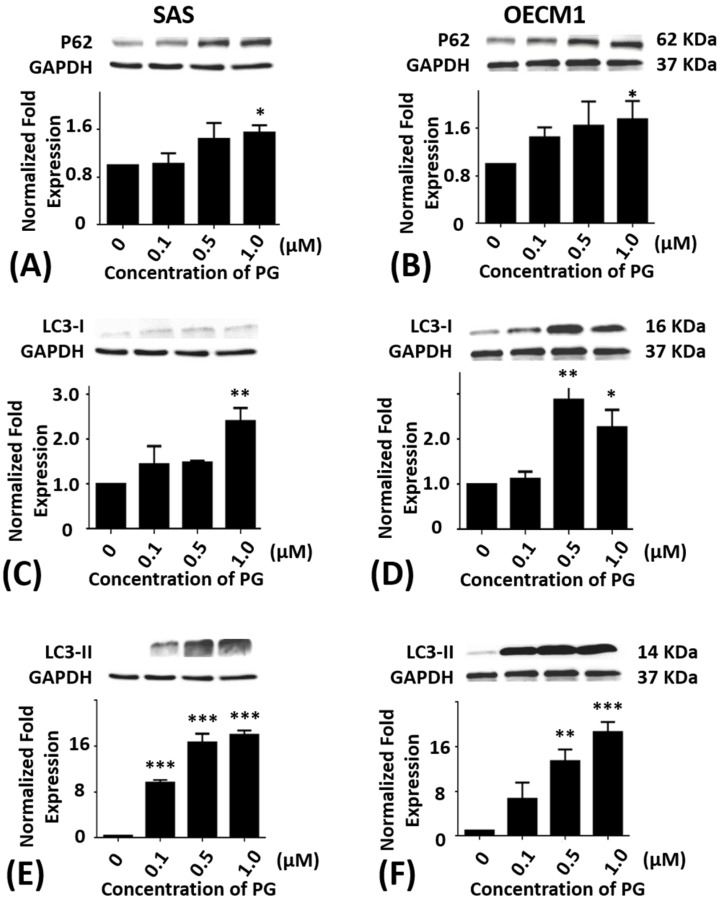
Altered protein levels of P62, LC3-I, and LC3-II of SAS and OECM1 cells treated with prodigiosin. SAS and OECM1 cells were treated with 0.1, 0.5, and 1.0 μM of prodigiosin (PG) for 24 h and lysed in RIPA buffer for Western blotting. Protein levels of P62 (**A**,**B**), LC3-I (**C**,**D**), and LC3-II (**E**,**F**) were shown as the mean ± SEM of three independent experiments. Protein levels were represented as ratio of band intensity to untreated control which were normalized via internal control GAPDH. * *P* < 0.05, ** *P* < 0.01, and *** *P* < 0.001 when compared with the untreated controls (0 μM).

Additionally, the P62 protein levels in SAS and OECM1 cells were significantly elevated in 1.0 μM of PG treatments as compared with the untreated controls (*P* < 0.05; Figure 5A,B). When compared with the untreated controls, the protein levels of LC3-I in SAS and OECM1 cells were elevated in 1.0 μM of PG treatments (*P* < 0.05; Figure 5C). The protein levels of LC3-I in OECM1 showed a significant increase in various concentrations of PG for 24 h treatment (*P* < 0.05; Figure 5D). The protein levels of LC3-II were markedly up-regulated in SAS cells with various concentrations of PG treatment for 24 h as compared with the untreated controls (*P* < 0.05; Figure 5E). While the protein levels of LC3-II also revealed a large increase in OECM1 cells with 0.5 and 1.0 μM of PG treatment for 24 h as compared with the untreated controls (*P* < 0.05; Figure 5F). These Western-blotting findings showed that autophagic cell death could be induced by PG treatment in oral cancer cells, which might occur through different signal pathways. Remarkably, the increase of P62, LC3-I, and LC3-II levels in the present study is associated with numerous investigations. Two carbazole alkaloids derived from *Murraya koenigii* (L.) Sprengel (Rutaceae) leaves, mahanine and isomahanine, resulted in increased accumulation of p62/sequestosome1 (p62/SQSTM1), with coordinated expression of LC3-II and cleaved caspase-3, suggesting inhibition of autophagic flux associated with carbazole alkaloid-induced apoptosis in the OSCC cell line CLS-354 [40]. Protein levels of LC3-II and p62 in human breast cancer cell lines MCF-7 and MDA-MB-231 are induced by ramalin that derived from the Antarctic lichen *Ramalina terebrata* [47]. One more recent report has demonstrated the autophagic cell death of HepG_2_ by dehydroepiandrosterone treatment and also via an increase of JNK-mediated P62 expression [48]. Nutrient depletion has augmented OSCC cell autophagy via increase of p62, LC3-II/LC3-I ratio, and GFP-LC3 levels in time-course patterns from 6 to 48 h when the inhibition of autophagy caused apoptosis in OSCC cells [49]. In study of anticancer effect of ursolic acid in apoptosis-resistant colorectal cancer, JNK signaling pathway has been triggered and further activated P62 expression [50]. Resveratrol can enhance autophagy via increase of JNK-mediated P62 expression and AMPK activation in chronic myelogenous leukemia cells [51,52]. Conversely, isomahanine, a carbazole alkaloid, obviously induces autophagic flux as shown by an increase in punctate GFP-LC3 and the LC3-II/LC3-I ratio with a concomitant p62 level decrease in multidrug-resistant human oral squamous cell carcinoma cells [53]. Treatment with grape seed extract and resveratrol in 4-nitroquinoline-1-oxide (4NQO)-induced tongue tumorigenesis of C57BL/6 mice, that decrease of autophagy flux marker p62 is observed [54]. Human lung cancer tissues that experienced chemotherapy shows an increase of LC3-I to LC3-II conversion and decrease of p62/sequestosome1 as compared with chemo-naïve cancer tissue as well as A549 cell [55]. Sunitinib, an oral multitargeted receptor tyrosine kinase inhibitor with antiangiogenic and antitumor activity that mainly targets vascular endothelial growth factor receptors, significantly increases the levels of LC3-II, concomitant with a decrease of p62 in rat pheochromocytoma PC12 cells [56]. Interestingly, the signaling of P62 seems to be a biphasic response for the induction of autophagy.

### 2.6. Phosphorylated Protein Levels of mTOR, Akt, and Ribosomal Protein S6 after PG Treatment

**Figure 6:**
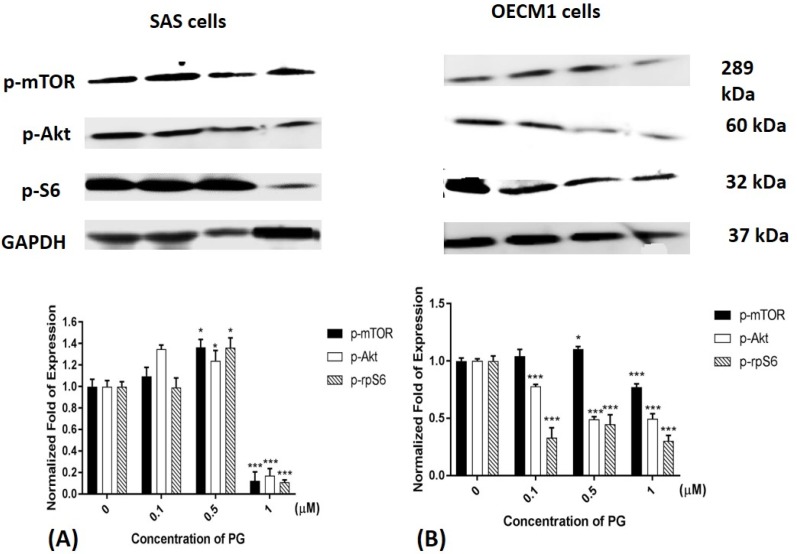
Altered protein levels of p-mTOR, p-Akt, and p-rpS6 of (**A**) SAS and (**B**) OECM1 cells treated with prodigiosin. SAS and OECM1 cells were treated with 0.1, 0.5, and 1.0 μM of prodigiosin (PG) for 24 h and lysed in RIPA buffer for Western blotting. Protein levels of p-mTOR, p-Akt, and p-rpS6 were shown as the mean ± SEM of three independent experiments. Protein levels were represented as ratio of band intensity to untreated control which were normalized via internal control GAPDH. * *P* < 0.05 and *** *P* < 0.001 when compared with the untreated controls (0 μM).

Previous studies have been shown that Akt and mTOR phosphorylation would be reduced while autophagy activation. Moreover, mTOR dephosphorylation reduced downstream protein p70S6K activation and finally inhibited ribosomal protein S6 (rpS6) phosphorylation [57,58,59]. Consequently, mTOR, Akt and rpS6 phosphorylation could be a marker of autophagy activation. After treatment of 1 μM PG, mTOR, Akt, and rpS6 phosphorylation in both SAS and OECM1 cells were significantly decreased (Figure 6). According to these results, PG could not only reduce mTOR and Akt protein expression, but also inhibit the phosphorylations of mTOR and Akt.

### 2.7. Effect of Prodigiosin on Autophagosome Formation in Oral Cancer Cells

**Figure 7:**
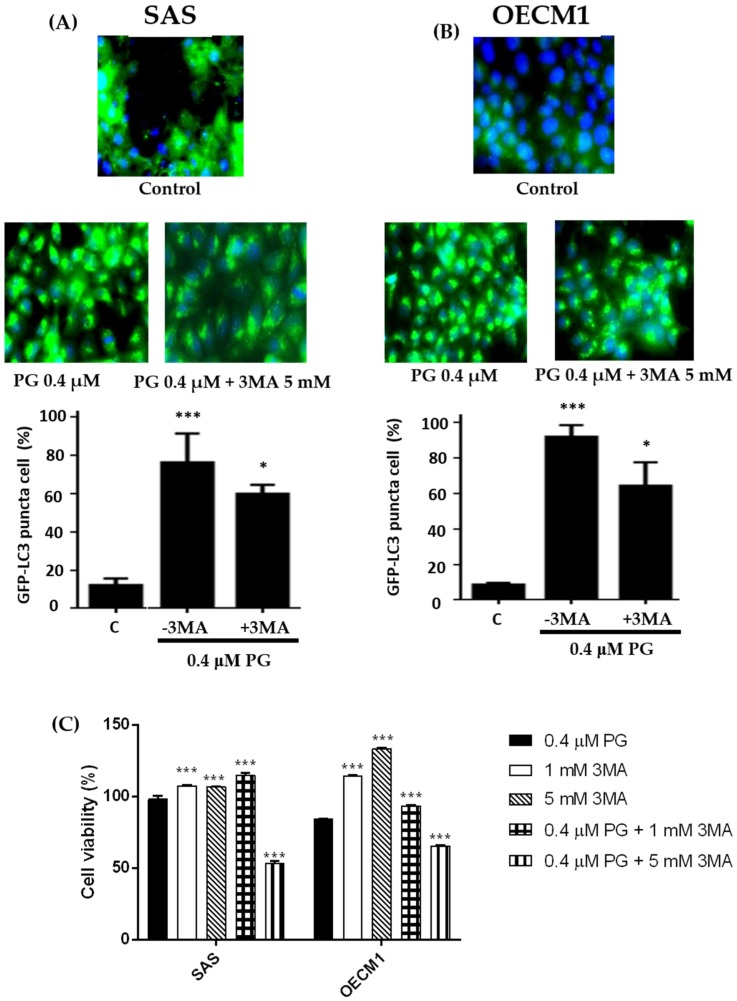
Alterations of autophosome formation of (**A**) SAS and (**B**) OECM1 cells treated with prodigiosin. SAS and OECM1 cells were treated with prodigiosin (PG; 0.1, 0.4, and 1.0 μM) and PG + 3MA (0.4 μM of PG and plus 5 mM of 3MA) for 24 h, followed by staining the autophagosome by LC3 antibody (green) and nucleus by DAPI (blue). The LC3 puncta cells of SAS and OECM1 were observed using fluorescence microscopy and counted the percentage of LC3 positive cell. (**C**) Cytotoxicity of treatment with 0.4 μM of PG + 3MA (1 and 5 mM) in two cell lines was observed by MTT assay. The results were shown as the mean ± SEM of three independent experiments. * *P* < 0.05 and *** *P* < 0.001 when compared with the untreated controls (0 μM).

For evaluating whether autophagy appeared in oral cancer cells after PG treatment, the expressions of LC3-II in autophagosome in SAS and OECM1 cells were further observed via immunofluorescence. During the cascade of autophagy signal pathways, LC3-II can anchor on the autophagosome, and its amount is correlated well with the numbers of autophagosomes [8,14]. Two oral cancer cells were incubated with 0.4 μM PG and 0.4 μM PG plus 5 mM of autophagic inhibitor 3-methyladenine (3MA) for 24 h when compared with the untreated control cells. The results illustrated that increased LC3 puncta cells were significantly increased by presenting numerous autophagosomes in SAS and OECM-1 treated with PG and decreased by the application of 3MA. Figure 7A,B showed the quantitative representations of autophagosome formation in SAS and OECM1 cells (*P* < 0.05). Furthermore, in order to prove PG-induced OSCC cells death was caused by autophagy, cell viability of two OSCC cells were tested after PG treatment with or without 1 mM and 5 mM of 3MA. The data illustrated that cell viability of PG combined with 1 mM 3MA in two cell lines were significantly higher than that of PG alone. However, cell viability of PG combined with 5 mM 3MA was significantly lower than that of PG alone. Meanwhile, 1 and 5 mM of 3MA could increase two cell lines growth (Figure 7C). Previous study has been demonstrated biphasic function of 3MA in cell autophagy. In nutrient-rich environment, 3MA would promote the autophagy [60], which might explain the decrease of cell viability after treatment of 5 mM 3MA plus PG. Take all above results together, autophagy indeed occurred in oral cancer cells in the induction of PG.

PG can down-regulate RAD51 expression, and trigger phosphorylation of JNK and p38 MAPK in many human breast carcinoma cell lines, which implicates the cytotoxicity of PG in this cancer [32]. The structural modification of the C-ring of prodigiosenes results in an anti-cancer activity of human K562 chronic myelogenous leukemia cells in both in vitro and in vivo assays [42]. From these data, PGs are thought to play critical roles in cancer therapy via inducing cell death [61]. Recently, PG can induce apoptosis in various cancer cells with low toxicity on normal cells, and PG-induced apoptosis may ascribe to Bcl-2 and survivin inhibition in colorectal cancer (HT-29) cells [62]. In addition, autophagy has been found in squamous cell carcinoma of the head and neck [63]. Increased protein levels of P62 and LC3-II in OSCC tissues have also been reported to correlate with survival, poor prognosis, and advanced stage cancer [64]. In this study, Western blot analyses showed that PG-induced autophagy in OECM1 cells by down-regulation of mTOR, PI3 kinase Class III, and Beclin-1 protein levels while by up-regulation of P62 and LC3 proteins. In SAS cells, PG-induced autophagy was associated with the involvement of decreased signals of mTOR, Akt, and Beclin-1 proteins while increase of P62 and LC3 proteins. Decreased protein level of cyclin D1 after 24 h treatment with 0.5 and 1.0 μM PG was observed in SAS and OECM1 cells (Figure 2), indicating G_0_/G_1_ checkpoint arrest. Furthermore, marked up-regulation of LC3-I and LC3-II protein levels was exhibited in OSCC cells, and a significant increase was found by presenting numerous autophagosomes in these cancer cells (Figure 7). Thereby, our findings provide the first evidence of increased autophagic signals were involved cell death by PG treatment in OECM1 and SAS cells. These results are consistent with previous findings for cell death of PG induction in melanoma cells of previous report. PG can be a specific mTOR inhibitor in melanoma cells, which might induce a loss of Akt phosphorylation, prevent its activation, and identify a possible new therapeutic option for this cancer [41]. Moreover, Akt is associated with cell survival and its expression could down-regulate the LC3-II expression; thereby, suppressing autophagy [11,12,13]. The PI3K Class III can be triggered through insulin and insulin-like receptors, for reduction of its signal to Akt; therefore, stimulating the protein level of mTOR, which is a central regulator of autophagy. Subsequently, the activation of mTOR would further suppress the autophagy pathway and induce protein synthesis. In fact, when mTOR is suppressed, autophagy is conversely induced. Autophagy is also promoted by AMP activated protein kinase (AMPK), which is a key energy sensor to maintain energy homeostasis [10].

## 3. Materials and Methods

### 3.1. Preparations of Prodigiosin

*Vibrio* sp. C1-TDSG02-1 was isolated from the sea sediment of Siaogang Harbor at a water depth of 17 m in eastern and southern Taiwan. The strain C1-TDSG02-1 was 99.0% identical with *Vibrio* sp. BL-182 (Genbank accession no. AY663829.1) based on 16S rDNA gene sequence. *Vibrio* sp. C1-TDSG02-1 was cultured in 1.4% soybean flour with 80% sterilized seawater at 25 °C, 5 Lpm (L/min), and pH 7.0–7.5 for 48 h. Extraction of the culture broth (8.0 L) with ethyl acetate (EtOAc, 4 × 8.0 L) yielded 45.7 g of crude extract. The EtOAc layer was separated on silica gel followed by elution chromatography with mixed *n*-hexane/EtOAc (stepwise, pure *n*-hexane, pure EtOAc) to yield 16 sub-fractions. Fraction 6 was chromatographed on silica and eluted using a mixture of *n*-hexane/acetone (stepwise, 10:1, pure acetone) to afford 11 sub-fractions. Sub-fraction 6-5 was chromatographed on silica and eluted using a mixture of *n*-hexane/acetone (4:1) to afford prodigiosin (PG, 1.94 g). The purity of purified PG is 95% confirmed by NMR. Prodigiosin (C_20_H_25_N_3_O; mol. wt.: 323.432 g/mole) was isolated as a red powder that gave an [M + H]^+^ ion peak at 324 *m*/*z* in the ESI/MS. One liter of culture can obtain approximately 1.398 g of PG.

### 3.2. Cell Cultures

Two human oral squamous carcinoma cell lines (SAS and OECM1) were obtained from Dr. Ta-Chun Yuan of Department of Life Science and Institute of Biotechnology (National Dong-Hwa University, Hualien, Taiwan). SAS were cultured in Dulbecco’s modified Eagle medium (DMEM, Thermo-Fisher, Waltham, MA, USA) supplemented with 5% fetal bovine serum (FBS, Thermo-Fisher), and 1% penicillin/streptomycin (PS, Thermo-Fisher). OECM1 were cultured in Roswell Park Memorial Institute medium 1640 (RPMI 1640) supplemented with 5% FBS and 1% PS. Two cell lines were cultured in CO_2_ incubator (Thermo-Fisher) and the culture condition was set up as 37 °C, 5% CO_2_. The medium was changed every 2 days, and the cells were detached by 0.25% trypsin/EDTA (Thermo-Fisher) for passage as reached 80–90% confluence. All experiments were obtained within 20 passages concerning uniformity and reproducibility. FL83B (mouse hepatocyte, ATCC CRL-2390) cells were maintained in Kaighn’s Modification of Ham’s F-12 Medium (F12K, Thermo-Fisher) supplemented with 10% FBS and 1% PS, and incubated at 37 °C with 5% CO_2_.

### 3.3. Cytotoxicity Assay

Cytotoxicity was measured by MTT assay. 7 × 10^3^ cells per well of SAS/OECM1 were seeded in 96-well plates and incubated at 37 °C, 5% CO_2_ overnight. Then, the cells were treated with various concentrations of PG (0.1, 0.5, 1.0, and 5.0 μM) for 24 h. After treatment, 20 μL per well of 50 mg/mL MTT (Thermo-Fisher) solution was added and incubated at 37 °C for 3 h. As incubation finished, all liquid in wells were replaced to dimethyl sulfoxide and the absorbance at 570 nm was measured by EnSpire Alpha plate reader (Perkin Elmer, Waltham, MA, USA). The absorbance at 570 nm was positively correlated to the number of viable cells so the cell viability was represented as the percentage of absorbance at 570 nm between treated and untreated cells.

### 3.4. Cell Cycle Analysis

A total of 7 × 10^4^ cells of SAS/OECM1 were seeded in 12-well plates and incubated at 37 °C, 5% CO_2_ overnight. Then, cells were incubated with 0.1, 0.5, and 1.0 μM of PG for 12 and 24 h. Next, cells were harvested by 0.25% trypsin/EDTA and fixed with 70% ethanol at −20 °C at least 1 h. The fixed cells were washed in cold phosphate buffer saline (PBS) twice, stained with 1 mL staining solution (20 μg/mL of propidium iodide (PI), 0.1% Triton X-100, 0.2 mg/mL RNase) at 37 °C for 30 min, and emission density at 617 nm was analyzed within 10^4^ cells for each treatment by Cytomics^TM^ FC500 flow cytometer (Beckman Coulter, Brea, CA, USA).

### 3.5. Western Blotting

A total of 7 × 10^4^ of OECM1 and SAS cell lines were seeded in 12-well plates and cells reached 80% confluence at 37 °C and 5% CO_2_. OECM1 and SAS cells were treated with 0.1, 0.5, and 1.0 μM of PG for 24 h. Then, media in wells were removed and washed twice with PBS. Cells in wells were homogenized using RIPA buffer and harvested into a 1.5 mL Eppendorf. The cell lysates were centrifuged at 12,000× *g* at 4 °C for 30 min, and the supernatant was kept at −20 °C until assayed. Interested proteins were separated using sodium dodecyl sulfate polyacrylamide gel electrophoresis and subsequently transferred to PVDF membrane (Millipore, Billerica, MA, USA). The membrane was blocked with 5% non-fat milk or 5% bovine serum albumin (for phosphorylated protein) in TBST saline (20 mM Tris-HCl, pH 7.4, 137 mM NaCl, and 0.05% Tween-20) at room temperature for 1 h, followed by incubation with an appropriate primary antibody at 4 °C overnight. The membrane was washed by TBST saline twice and then incubated with peroxidase conjugated secondary antibody for 1 h. Finally, the membrane was rinsed with ECL reagent (Amershan Bioscience, Little Chalfont, UK) for 1 min and chemiluminescence was collected with a LAS-3000 imager (Fujifilm, Tokyo, Japan). GAPDH was taken as an internal control for normalization. Table 3 shows primary and secondary antibodies used in this study.

### 3.6. Autophagosome Formation Analysis

OECM1 and SAS cells were plated into 96-well plates at a cell density of 5 × 10^3^/well. The cells were incubated at 37 °C and 5% CO_2_. OECM1 and SAS cells were incubated in 0.4 μM of PG and 0.4 μM of PG plus 5 mM of 3-methyladenine (3MA) for 24 h. Then, cells were fixed with 3.7% formaldehyde/PBS and stained by the Alexa Fluor 488-conjugated anti-LC3-II rabbit antibody (Thermo-Fisher). Images were captured by a Typhoon™ FLA 9000 Biomolecular Imager (GE Healthcare, Little Chalfont, UK). The fluorescence focus units were quantified in each well.

### 3.7. Statistical Analysis

Data were expressed as mean ± SEM of at least three independent experiments. The results were analyzed by one-way analysis of variance (ANOVA) followed by a Tukey’s test. Significant differences (*P* < 0.05) between the means of control and treatment were analyzed. All statistical procedures were performed with GraphPad Prism Ver 5.0 software (GraphPad Software, La Jolla, San Diego, CA, USA).

## 4. Conclusions

**Figure 8:**
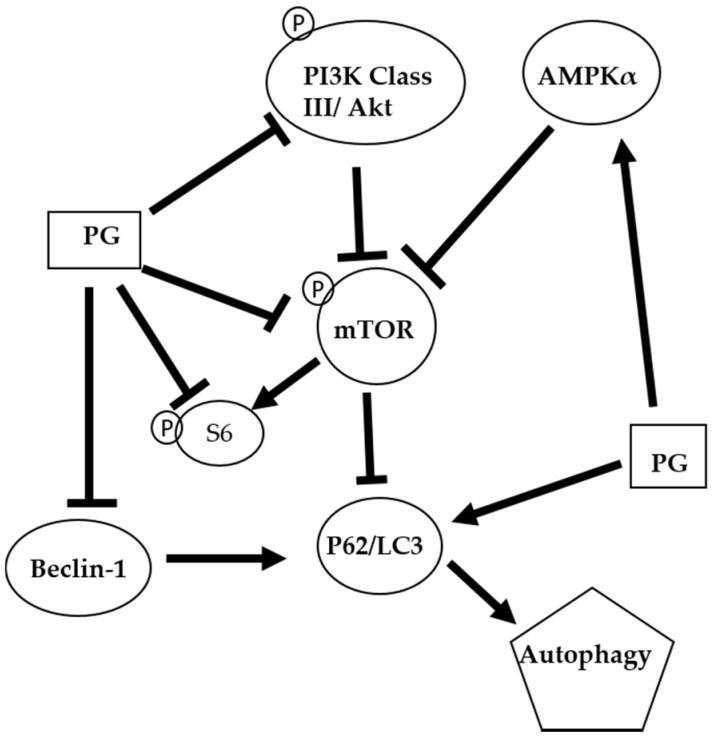
The proposed autophagic mechanism of prodigiosin against oral cancer cells. AMP-activated protein kinase alpha (AMPKα); Mammalian target of rapamycin (mTOR); Microtubule-associated protein 1A/1B-light chain 3 (LC3); Nucleoporin 62 (P62); Phosphoinositide 3-kinase (PI3K); Prodigiosin (PG); Protein kinase B (Akt); and Ribosomal protein S6 (S6)

The autophagic mechanism of PG against oral cancer cells was proposed (Figure 8). PG might inhibit cell growth via suppressing the cyclin D1 to cause the arresting cell cycle in G_0_/G_1_ phase. Furthermore, PG could mediate AMPKα, PI3K class III/Akt signal pathway and directly or indirectly exerts the inhibition of mTOR and Beclin-1 and the induction of p62/LC3 resulting in cell autophagy. In the present study revealed that PG could induce autophagic cell death in human oral cancer cells by LC3-mediated P62/LC3-I/LC3-II pathway in vitro. Our findings elucidated the inhibitory role of PG in this OSCC cancer, which may target the autophagic pathways as a potential agent in cancer therapeutics. Further work in studying anticancer activity of PG should focus on the in vivo test of PG. In a lung cancer xenograft model in vivo studies have demonstrated cancer growth inhibition of tumor growth via cell apoptosis and invasion. Moreover, PG has inhibited RhoA and MMP-2 protein expression in lung cancer 95-D cell line resulting in invasion inhibition [65]. In addition, the activation of p73 and c-Jun-mediated ΔNp73 signaling pathway by PG induced, which can restore p53 tumor suppressor activity in colon cancer [28,66]. In breast cancer, PG could downregulate the Wnt/β-catenin signaling pathway, resulting in the triggered apoptosis process [67]. Based on the present study, this is the first evident that (1) autophagy-induced activity of PG; and (2) growth inhibiting activity of PG in OSCC. This study has confirmed that PG might have a chemotherapeutic potential and promise in treating oral squamous cell carcinoma.

## Figures and Tables

**Table 1:** Prodigiosin mediated cell cycle distribution in SAS cells.

Cell	Dosage (μM)	Sub G_1_ (%)	G_0_/G_1_ (%)	S (%)	G_2_/M (%)
12 h	0	2.2 ± 0.8	40.3 ± 3.3	25.2 ± 4.4	32.4 ± 2.9
	0.1	1.7 ± 0.8	45.3 ± 4.1 *	23.2 ± 0.2	29.9 ± 0.9
	0.5	1.0 ± 0.2	51.2 ± 1.9 *	21.1 ± 2.8	26.6 ± 0.7 *
	1.0	1.4 ± 0.7	51.4 ± 1.2 *	20.0 ± 2.7	27.2 ± 0.2 *
24 h	0	0.9 ± 0.3	42.1 ± 2.7	20.4 ± 2.7	36.6 ± 2.1
	0.1	1.4 ± 0.2	39.7 ± 2.2	25.0 ± 2.0	33.9 ± 3.6
	0.5	1.7 ± 0.3 *	51.7 ± 3.2 *	20.9 ± 0.8	25.7 ± 2.5 *
	1.0	2.5 ± 0.7 *	54.0 ± 3.7 *	17.0 ± 0.7	26.3 ± 3.2 *

SAS cells were treated with 0.1, 0.5, and 1.0 μM of prodigiosin (PG) for 12 and 24 h, respectively, and stained with propidium iodide. The results are shown as the mean ± SEM of three independent experiments. * *P* < 0.05, compared with the untreated control (0 μM).

**Table 2:** Prodigiosin mediated cell cycle distribution in OECM1 cells.

Cell	Dosage (μM)	Sub G_1_ (%)	G_0_/G_1_ (%)	S (%)	G_2_/M (%)
12 h	0	0.5 ± 0.1	50.9 ± 1.7	16.6 ± 1.0	32.1 ± 0.4
	0.1	0.5 ± 0.2	52.3 ± 0.5	17.0 ± 0.5	30.2 ± 2.2
	0.5	0.4 ± 0.1	63.2 ± 0.6 **	12.2 ± 0.2 *	24.1 ± 1.3 *
	1.0	0.4 ± 0.1	63.3 ± 0.4 **	10.5 ± 0.2 *	25.7 ± 0.8 *
24 h	0	1.2 ± 0.2	47.9 ± 2.3	14.0 ± 1.6	36.9 ± 3.1
	0.1	1.1 ± 0.1	50.9 ± 3.8	21.9 ± 2.9 *	26.1 ± 1.6 *
	0.5	1.4 ± 0.1	60.2 ± 2.5 *	19.8 ± 3.1 *	18.7 ± 2.3 **
	1.0	1.2 ± 0.1	61.8 ± 0.4 *	18.4 ± 2.6 *	18.7 ± 3.3 **

OECM1 cells were treated with 0.1, 0.5, and 1.0 μM of prodigiosin (PG) for 12 and 24 h, respectively, and stained with propidium iodide. The results are shown as the mean ± SEM of three independent experiments. * *P* < 0.05 and ** *P* < 0.01, compared with the untreated control (0 μM).

**Table 3:** Primary and second antibodies used in the study.

Antibody	MW(kDa)	Dilution	Sources
mTOR	289	1:1000	Cell Signalling
p-mTOR (Ser2448)	289	1:200	Santa Cruz
PI3K class III	100	1:1000	Cell Signalling
AMPKα	62	1:1000	Cell Signalling
P62	62	1:1000	Cell Signalling
Akt	60	1:1000	Cell Signalling
P-Akt (Ser473)	60	1:200	Santa Cruz
Beclin-1	60	1:1000	Cell Signalling
Cyclin D1	34	1:1000	Cell Signalling
p-Ribosomal protein S6 (Ser235/236)	32	1:200	Santa Cruz
LC3-I	16	1:1000	Cell Signalling
LC3-II	14	1:1000	Cell Signalling
GADPH	37	1:10,000	Cell Signalling
anti-Rabbit (IgG)	-	1:5000	GeneTex
anti-Mouse (IgG)	-	1:10,000	GE

## References

[1] Marques L.A., Eluf-Neto J., Figueiredo R.A., Gois-Filho J.F., Kowalski L.P., Carvalho M.B., Abrahao M., Wunsch-Filho V. (2008). Oral health, hygiene practices and oral cancer. Rev. Saude Publica.

[2] Sciubba J.J. (2001). Oral cancer. The importance of early diagnosis and treatment. Am. J. Clin. Dermatol..

[3] Olsen S.M., Moore E.J., Koch C.A., Kasperbauer J.L., Olsen K.D. (2011). Oral cavity and oropharynx squamous cell carcinoma with metastasis to the parotid lymph nodes. Oral Oncol..

[4] Singletary K., Milner J. (2008). Diet, autophagy, and cancer: A review. Cancer Epidemiol. Biomark. Prev..

[5] Wu J.J., Quijano C., Chen E., Liu H., Cao L., Fergusson M.M., Rovira I.I., Gutkind S., Daniels M.P., Komatsu M. (2009). Mitochondrial dysfunction and oxidative stress mediate the physiological impairment induced by the disruption of autophagy. Aging.

[6] Luo G.X., Cai J., Lin J.Z., Luo W.S., Luo H.S., Jiang Y.Y., Zhang Y. (2012). Autophagy inhibition promotes gambogic acid-induced suppression of growth and apoptosis in glioblastoma cells. Asian Pac. J. Cancer Prev..

[7] Shinojima N., Yokoyama T., Kondo Y., Kondo S. (2007). Roles of the akt/mtor/p70s6k and erk1/2 signaling pathways in curcumin-induced autophagy. Autophagy.

[8] Han H.Y., Kim H., Jeong S.H., Lim D.S., Ryu M.H. (2014). Sulfasalazine induces autophagic cell death in oral cancer cells via akt and erk pathways. Asian Pac. J. Cancer Prev..

[9] Ellington A.A., Berhow M.A., Singletary K.W. (2006). Inhibition of akt signaling and enhanced erk1/2 activity are involved in induction of macroautophagy by triterpenoid b-group soyasaponins in colon cancer cells. Carcinogenesis.

[10] Yao F., Lv Y.C., Zhang M., Xie W., Tan Y.L., Gong D., Cheng H.P., Liu D., Li L., Liu X.Y. (2015). Apelin-13 impedes foam cell formation by activating class iii pi3k/beclin-1-mediated autophagic pathway. Biochem. Biophys. Res. Commun..

[11] Sarbassov D.D., Guertin D.A., Ali S.M., Sabatini D.M. (2005). Phosphorylation and regulation of akt/pkb by the rictor-mtor complex. Science.

[12] Degtyarev M., De Maziere A., Orr C., Lin J., Lee B.B., Tien J.Y., Prior W.W., van Dijk S., Wu H., Gray D.C. (2008). Akt inhibition promotes autophagy and sensitizes pten-null tumors to lysosomotropic agents. J. Cell Biol..

[13] Roy B., Pattanaik A.K., Das J., Bhutia S.K., Behera B., Singh P., Maiti T.K. (2014). Role of pi3k/akt/mtor and mek/erk pathway in concanavalin a induced autophagy in hela cells. Chem. Biol. Interact..

[14] Song L., Ma L., Chen G., Huang Y., Sun X., Jiang C., Liu H. (2016). Autophagy inhibitor 3-methyladenine enhances the sensitivity of nasopharyngeal carcinoma cells to chemotherapy and radiotherapy. Zhong Nan Da Xue Xue Bao Yi Xue Ban.

[15] Chang C.C., Chen W.C., Ho T.F., Wu H.S., Wei Y.H. (2011). Development of natural anti-tumor drugs by microorganisms. J. Biosci. Bioeng..

[16] Marchal E., Smithen D.A., Uddin M.I., Robertson A.W., Jakeman D.L., Mollard V., Goodman C.D., MacDougall K.S., McFarland S.A., McFadden G.I. (2014). Synthesis and antimalarial activity of prodigiosenes. Org. Biomol. Chem..

[17] Lapenda J.C., Silva P.A., Vicalvi M.C., Sena K.X., Nascimento S.C. (2015). Antimicrobial activity of prodigiosin isolated from serratia marcescens ufpeda 398. World J. Microbiol. Biotechnol..

[18] Wang Y., Nakajima A., Hosokawa K., Soliev A.B., Osaka I., Arakawa R., Enomoto K. (2012). Cytotoxic prodigiosin family pigments from *pseudoalteromonas sp.* 1020r isolated from the pacific coast of Japan. Biosci. Biotechnol. Biochem..

[19] Kimyon O., Das T., Ibugo A.I., Kutty S.K., Ho K.K., Tebben J., Kumar N., Manefield M. (2016). Serratia secondary metabolite prodigiosin inhibits pseudomonas aeruginosa biofilm development by producing reactive oxygen species that damage biological molecules. Front. Microbiol..

[20] Song Y., Liu G., Li J., Huang H., Zhang X., Zhang H., Ju J. (2015). Cytotoxic and antibacterial angucycline- and prodigiosin-analogues from the deep-sea derived *streptomyces sp.* Scsio 11594. Mar. Drugs.

[21] Kancharla P., Lu W., Salem S.M., Kelly J.X., Reynolds K.A. (2014). Stereospecific synthesis of 23-hydroxyundecylprodiginines and analogues and conversion to antimalarial premarineosins via a rieske oxygenase catalyzed bicyclization. J. Org. Chem..

[22] Perez-Tomas R., Vinas M. (2010). New insights on the antitumoral properties of prodiginines. Curr. Med. Chem..

[23] Sam S., Sam M.R., Esmaeillou M., Safaralizadeh R. (2016). Effective targeting survivin, caspase-3 and microrna-16–1 expression by methyl-3-pentyl-6-methoxyprodigiosene triggers apoptosis in colorectal cancer stem-like cells. Pathol. Oncol. Res..

[24] Yu C.J., Ou J.H., Wang M.L., Jialielihan N., Liu Y.H. (2015). Elevated survivin mediated multidrug resistance and reduced apoptosis in breast cancer stem cells. J. BUON.

[25] Papireddy K., Smilkstein M., Kelly J.X., Shweta, Salem S.M., Alhamadsheh M., Haynes S.W., Challis G.L., Reynolds K.A. (2011). Antimalarial activity of natural and synthetic prodiginines. J. Med. Chem..

[26] Chang C.C., Wang Y.H., Chern C.M., Liou K.T., Hou Y.C., Peng Y.T., Shen Y.C. (2011). Prodigiosin inhibits gp91(phox) and inos expression to protect mice against the oxidative/nitrosative brain injury induced by hypoxia-ischemia. Toxicol. Appl. Pharmacol..

[27] Dalili D., Fouladdel S., Rastkari N., Samadi N., Ahmadkhaniha R., Ardavan A., Azizi E. (2012). Prodigiosin, the red pigment of *serratia marcescens*, shows cytotoxic effects and apoptosis induction in ht-29 and t47d cancer cell lines. Nat. Prod. Res..

[28] Hong B., Prabhu V.V., Zhang S., van den Heuvel A.P., Dicker D.T., Kopelovich L., El-Deiry W.S. (2014). Prodigiosin rescues deficient p53 signaling and antitumor effects via upregulating p73 and disrupting its interaction with mutant p53. Cancer Res..

[29] Krishna P.S., Vani K., Prasad M.R., Samatha B., Bindu N.S., Charya M.A., Reddy Shetty P. (2013). In silico molecular docking analysis of prodigiosin and cycloprodigiosin as cox-2 inhibitors. Springerplus.

[30] Hosseini A., Espona-Fiedler M., Soto-Cerrato V., Quesada R., Perez-Tomas R., Guallar V. (2013). Molecular interactions of prodiginines with the bh3 domain of anti-apoptotic bcl-2 family members. PLoS ONE.

[31] Elahian F., Moghimi B., Dinmohammadi F., Ghamghami M., Hamidi M., Mirzaei S.A. (2013). The anticancer agent prodigiosin is not a multidrug resistance protein substrate. DNA Cell Biol..

[32] Pan M.Y., Shen Y.C., Lu C.H., Yang S.Y., Ho T.F., Peng Y.T., Chang C.C. (2012). Prodigiosin activates endoplasmic reticulum stress cell death pathway in human breast carcinoma cell lines. Toxicol. Appl. Pharmacol..

[33] Chang Y.T., Huang C.Y., Li K.T., Li R.N., Liaw C.C., Wu S.H., Liu J.R., Sheu J.H., Chang H.W. (2016). Sinuleptolide inhibits proliferation of oral cancer ca9–22 cells involving apoptosis, oxidative stress, and DNA damage. Arch. Oral Biol..

[34] Dai W., Sun C., Huang S., Zhou Q. (2016). Carvacrol suppresses proliferation and invasion in human oral squamous cell carcinoma. Onco Targets Ther..

[35] Kim D.J., Lee J.H., Park H.R., Choi Y.W. (2016). Acetylshikonin inhibits growth of oral squamous cell carcinoma by inducing apoptosis. Arch. Oral Biol..

[36] Liu C.M., Peng C.Y., Liao Y.W., Lu M.Y., Tsai M.L., Yeh J.C., Yu C.H., Yu C.C. (2017). Sulforaphane targets cancer stemness and tumor initiating properties in oral squamous cell carcinomas via mir-200c induction. J. Formos. Med. Assoc..

[37] Su N.W., Wu S.H., Chi C.W., Liu C.J., Tsai T.H., Chen Y.J. (2017). Metronomic cordycepin therapy prolongs survival of oral cancer-bearing mice and inhibits epithelial-mesenchymal transition. Molecules.

[38] Kwak H.H., Kim I.R., Kim H.J., Park B.S., Yu S.B. (2016). Alpha-mangostin induces apoptosis and cell cycle arrest in oral squamous cell carcinoma cell. Evid. Based Complement. Altern. Med..

[39] Tjioe K.C., Tostes Oliveira D., Gavard J. (2016). Luteolin impacts on the DNA damage pathway in oral squamous cell carcinoma. Nutr. Cancer.

[40] Utaipan T., Athipornchai A., Suksamrarn A., Jirachotikoon C., Yuan X., Lertcanawanichakul M., Chunglok W. (2017). Carbazole alkaloids from murraya koenigii trigger apoptosis and autophagic flux inhibition in human oral squamous cell carcinoma cells. J. Nat. Med..

[41] Espona-Fiedler M., Soto-Cerrato V., Hosseini A., Lizcano J.M., Guallar V., Quesada R., Gao T., Perez-Tomas R. (2012). Identification of dual mtorc1 and mtorc2 inhibitors in melanoma cells: Prodigiosin vs. Obatoclax. Biochem. Pharmacol..

[42] Smithen D.A., Forrester A.M., Corkery D.P., Dellaire G., Colpitts J., McFarland S.A., Berman J.N., Thompson A. (2013). Investigations regarding the utility of prodigiosenes to treat leukemia. Org. Biomol. Chem..

[43] Alexandrova E.M., Yallowitz A.R., Li D., Xu S., Schulz R., Proia D.A., Lozano G., Dobbelstein M., Moll U.M. (2015). Improving survival by exploiting tumour dependence on stabilized mutant p53 for treatment. Nature.

[44] Kim J., Kundu M., Viollet B., Guan K.L. (2011). Ampk and mtor regulate autophagy through direct phosphorylation of ulk1. Nat. Cell Biol..

[45] Mihaylova M.M., Shaw R.J. (2011). The ampk signalling pathway coordinates cell growth, autophagy and metabolism. Nat. Cell Biol..

[46] Funderburk S.F., Wang Q.J., Yue Z. (2010). The beclin 1-vps34 complex—At the crossroads of autophagy and beyond. Trends Cell Biol..

[47] Lee E., Lee C.G., Yim J.H., Lee H.K., Pyo S. (2016). Ramalin-mediated apoptosis is enhanced by autophagy inhibition in human breast cancer cells. Phytother. Res..

[48] Vegliante R., Desideri E., Di Leo L., Ciriolo M.R. (2016). Dehydroepiandrosterone triggers autophagic cell death in human hepatoma cell line hepg2 via jnk-mediated p62/sqstm1 expression. Carcinogenesis.

[49] Jiang L.C., Xin Z.Y., Deborah B., Zhang J.S., Yuan D.Y., Xu K., Liu X.B., Jiang H.Q., Fan Q.C., Zhang B. (2015). Inhibition of autophagy augments apoptosis in human oral squamous cell carcinoma under nutrient depletion. J. Oral Pathol. Med..

[50] Xavier C.P., Lima C.F., Pedro D.F., Wilson J.M., Kristiansen K., Pereira-Wilson C. (2013). Ursolic acid induces cell death and modulates autophagy through jnk pathway in apoptosis-resistant colorectal cancer cells. J. Nutr. Biochem..

[51] Puissant A., Auberger P. (2010). Ampk- and p62/sqstm1-dependent autophagy mediate resveratrol-induced cell death in chronic myelogenous leukemia. Autophagy.

[52] Puissant A., Robert G., Fenouille N., Luciano F., Cassuto J.P., Raynaud S., Auberger P. (2010). Resveratrol promotes autophagic cell death in chronic myelogenous leukemia cells via jnk-mediated p62/sqstm1 expression and ampk activation. Cancer Res..

[53] Utaipan T., Athipornchai A., Suksamrarn A., Chunsrivirot S., Chunglok W. (2017). Isomahanine induces endoplasmic reticulum stress and simultaneously triggers p38 mapk-mediated apoptosis and autophagy in multidrug-resistant human oral squamous cell carcinoma cells. Oncol. Rep..

[54] Shrotriya S., Tyagi A., Deep G., Orlicky D.J., Wisell J., Wang X.J., Sclafani R.A., Agarwal R., Agarwal C. (2015). Grape seed extract and resveratrol prevent 4-nitroquinoline 1-oxide induced oral tumorigenesis in mice by modulating ampk activation and associated biological responses. Mol. Carcinog..

[55] Lee J.G., Shin J.H., Shim H.S., Lee C.Y., Kim D.J., Kim Y.S., Chung K.Y. (2015). Autophagy contributes to the chemo-resistance of non-small cell lung cancer in hypoxic conditions. Respir. Res..

[56] Ikeda T., Ishii K.A., Saito Y., Miura M., Otagiri A., Kawakami Y., Shimano H., Hara H., Takekoshi K. (2013). Inhibition of autophagy enhances sunitinib-induced cytotoxicity in rat pheochromocytoma pc12 cells. J. Pharmacol. Sci..

[57] Huang S. (2013). Inhibition of pi3k/akt/mtor signaling by natural products. Anticancer Agents Med. Chem..

[58] Park D., Jeong H., Lee M.N., Koh A., Kwon O., Yang Y.R., Noh J., Suh P.-G., Park H., Ryu S.H. (2016). Resveratrol induces autophagy by directly inhibiting mtor through atp competition. Sci. Rep..

[59] Din F.V.N., Valanciute A., Houde V.P., Zibrova D., Green K.A., Sakamoto K., Alessi D.R., Dunlop M.G. (2012). Aspirin inhibits mtor signaling, activates amp-activated protein kinase, and induces autophagy in colorectal cancer cells. Gastroenterology.

[60] Wu Y.-T., Tan H.-L., Shui G., Bauvy C., Huang Q., Wenk M.R., Ong C.-N., Codogno P., Shen H.-M. (2010). Dual role of 3-methyladenine in modulation of autophagy via different temporal patterns of inhibition on class i and iii phosphoinositide 3-kinase. J. Biol. Chem..

[61] White E., Karp C., Strohecker A.M., Guo Y., Mathew R. (2010). Role of autophagy in suppression of inflammation and cancer. Curr. Opin. Cell Biol..

[62] Hassankhani R., Sam M.R., Esmaeilou M., Ahangar P. (2015). Prodigiosin isolated from cell wall of *serratia marcescens* alters expression of apoptosis-related genes and increases apoptosis in colorectal cancer cells. Med. Oncol..

[63] Cosway B., Lovat P. (2016). The role of autophagy in squamous cell carcinoma of the head and neck. Oral Oncol..

[64] Liu J.L., Chen F.F., Lung J., Lo C.H., Lee F.H., Lu Y.C., Hung C.H. (2014). Prognostic significance of p62/sqstm1 subcellular localization and lc3b in oral squamous cell carcinoma. Br. J. Cancer.

[65] Zhang J., Shen Y., Liu J., Wei D. (2005). Antimetastatic effect of prodigiosin through inhibition of tumor invasion. Biochem. Pharmacol..

[66] Prabhu V.V., Hong B., Allen J.E., Zhang S., Lulla A.R., Dicker D.T., El-Deiry W.S. (2016). Small-molecule prodigiosin restores p53 tumor suppressor activity in chemoresistant colorectal cancer stem cells via c-jun-mediated δnp73 inhibition and p73 activation. Cancer Res..

[67] Wang Z., Li B., Zhou L., Yu S., Su Z., Song J., Sun Q., Sha O., Wang X., Jiang W. (2016). Prodigiosin inhibits wnt/β-catenin signaling and exerts anticancer activity in breast cancer cells. Proc. Natl. Acad. Sci. USA.

